# Successful use of long acting octreotide in two cases with Beckwith-Wiedemann syndrome and severe hypoglycemia

**DOI:** 10.1186/1687-9856-2014-18

**Published:** 2014-09-15

**Authors:** Hiba Al-Zubeidi, Michael E Gottschalk, Ron S Newfield

**Affiliations:** 1 Pediatrics, Rady Children's Hospital San Diego, MC5103, 3020 Children’s Way, San Diego, CA 92123-4282, USA; 2 Pediatrics, University of California, San Diego, San Diego, CA, USA

**Keywords:** Hypoglycemia, Hyperinsulinism, Beckwith-Wiedemann syndrome

## Abstract

**Introduction:**

Hyperinsulinism associated with Beckwith-Wiedemann syndrome (BWS) can occur in about 50% of cases, causing hypoglycemia of variable severity. Parenteral use of octreotide may be indicated if unresponsive to diazoxide. There is limited data on use of octreotide in BWS.

**Objective:**

Chart review describing 2 cases with BWS and hypoglycemia treated with long acting Octreotide as a monthly injection.

**Cases:**

We describe two unrelated females born large for gestational age found to have clinical features consistent with BWS, who developed severe hypoglycemia. Genetic diagnosis of BWS was confirmed. The first patient was born at 37 weeks and developed hypoglycemia shortly after birth. She was initially started on diazoxide but developed pulmonary congestion and was therefore switched to depot octreotide (LAR). She maintained euglycemia with LAR. In the second patient (born at 26-4/7 weeks), onset of hypoglycemia was delayed till 11 weeks of age due to hydrocortisone (indicated hemodynamically) and continuous feeding, and was partially responsive to diazoxide. She was switched to octreotide 4 times daily, treated till at age 18 months. Despite frequent feeds, she required treatment again between ages 4–6.5 years, initially with diazoxide but due to severe hypertrichosis she was switched to LAR with an excellent response. Both patients treated with LAR for over two years achieved euglycemia above 70 mg/dl and had normal height gain, without side effects.

**Conclusion:**

Successful treatment of hypoglycemia can be achieved and maintained with LAR in infants and children with BWS who are either resistant or cannot tolerate diazoxide.

## Background

Beckwith-Wiedemann syndrome (BWS) is a genetic disorder and is the most common overgrowth syndrome in infancy with an incidence of 1:13,700 births. It is characterized by commonly having one or more of the following: macrosomia, macroglossia, hemihypertrophy, omphalocele, ear creases/pits and predisposition for childhood tumors [1]. Hyperinsulinism associated with BWS can occur in about 50% of cases, causing hypoglycemia of variable severity [2]. In 20% it can be more severe and prolonged [3,4]. BWS is associated with abnormal regulation of gene transcription in 2 imprinted domains on chromosome 11p15.5. Regulation may be disrupted by numerous mechanisms [5,6]. While the different genetic defects causing BWS have been correlated with the risk of tumor genesis and some dysmorphic features there has been no significant differences between BWS molecular subgroups and the frequencies of neonatal hypoglycemia [5,7]. It is important to identify and treat the hyperinsulinemic hypoglycemia in this condition early and aggressively, in order to prevent adverse neurological outcomes and intellectual impairment [4]. There is limited literature on the use of long acting octreotide for the treatment of neonatal hypoglycemia in general [8,9]. To our knowledge, there are no known reports of the use of LAR depot octreotide (LAR) in the treatment of hypoglycemia due to BWS. There is one case report of the short-acting form of octreotide given multiple times daily, used successfully in a child with BWS [10].

We describe two cases successfully treated long term with LAR. We propose that the parenteral use of octreotide may be indicated if patients are unresponsive to diazoxide or develop undesirable side effects.

## Case presentations

### Patient 1

A female infant of Hispanic descent was born large for gestational age (LGA) at 4.61 kg at 37 weeks gestation to a 31-year-old mother. Pregnancy was uncomplicated except for fetal macrosomia. At birth she had clinical features consistent with BWS that included macroglossia, hemihypertrophy, small omphalocele and high α-fetoprotein (36300 ng/ml). Genetic testing showed L1T1 (KCNQ1OT1) hypomethylation and H19 hypermethylation. On day of life (DOL) 1 she developed severe hypoglycemia, requiring continuous glucose infusion. While she was on frequent feeds she had severe hypoglycemia with relative hyperinsulinism: nadir glucose 10 mg/dl, insulin 2 uIU/mL, β-hydroxybutarate 1.2 mg/dl, and noncontributory cortisol and growth hormone levels. Her brisk glucose rise in response to glucagon was supportive of hyperinsulinism as well. She was started on diazoxide for persistent hypoglycemia on DOL10 and despite very high doses, up to 22 mg/kg/day she remained hypoglycemic, and developed pulmonary congestion. She was then switched to sc octreotide every 8 hours, starting at 15 mcg/kg/day and was titrated up to 40 mcg/kg/day. She was converted a week later to LAR starting at 6 mg monthly, which was 30 times her daily dose that kept her glucose steady. However, she was not fully controlled with her hypoglycemia on that initial LAR dose, and she received an extra dose of 3 mg LAR a few days later. Following that she received 9 mg every month and remained euglycemic past the age of 2 years old, when we felt we can lower her dose of LAR gradually. She is currently 3 years old and maintains euglycemia on a monthly dose of 7 mg of LAR and tolerates 8 hours or longer of overnight fast.

Her high α-fetoprotein trended down over 12 months (36300 to 20 ng/ml). Having being monitored with frequent ultrasounds, she was diagnosed with Wilms tumor stage I favorable histology at age 2 and underwent a right lower partial nephrectomy and had received chemotherapy without any complications.

Patient didn’t develop any side effects from the LAR and maintained normal linear growth and weight; IGF-1 was normal prior to treatment and she continued to grow well with her height at the 75th percentile at the beginning of treatment and she remained at that percentile and above, therefore IGF1 level was not repeated. She had no gallstones or sludge.

### Patient 2

A female infant of Hispanic descent was born LGA at 1.46 Kg at 26-4/7 weeks to a 32-year-old mother. Neonatal course complicated by tracheostomy secondary to macroglossia, hemodynamic instability and hypotension requiring daily hydrocortisone replacement at 15 mg/m2/day, transient primary hypothyroidism with TSH over 300 uIU/mL likely due to iodine exposure during NICU stay when she underwent PDA closure, and elevated α-fetoprotein. Genetic testing showed variant BWS due to unbalanced translocation with partial trisomy 11P and partial monsomy of chromosome 8. The daily hydrocortisone (HC) together with continuous nasogastric feeding, prevented her from becoming hypoglycemic initially but once she was transitioned, at age 11 weeks of life, off of continuous nasogastric feedings, she developed severe hypoglycemia right away, and to address this, initially her HC dose was increased to 20 mg/m2/day**.** She had relative hyperinsulinemia: glucose 10 mg/dl, insulin level 1uIU/ml, β-hydroxybutyrate 0.6 mg/dl and cortisol 2.3 mcg/dl. The cortisol levels were drawn as part of the critical sample for hypoglycemia evaluation and the patient was already receiving HC. The low level represents adrenal insufficiency secondary to suppressed ACTH-adrenal axis due to high dose hydrocortisone treatment. The HC was tapered gradually and was discontinued at age 11 months. She didn’t exhibit any clinical signs of adrenal insufficiency since then. She remained hypoglycemic on a relatively high dose of hydrocortisone of up to 20 mg/m2/day. She was only partially responsive to diazoxide, so she was switched to sc octreotide, and was kept euglycemic (blood glucose values over 70 mg/dl) on 19 mcg/kg/day given in 4 daily doses. She continued to receive Octreotide and low dose levothyroxine of 25 mcg/day, till she was 18 months of age, when she was tried off both. She remained euglycemic on frequent feeds given every 2 hours during the day and overnight feeds running at 50 ml/hr for 10 hours with 8 tablespoons of cornstarch. She remained euthyroid, but she had developed excessive weight gain on this feeding regimen and an attempt to decrease her frequency of feeds and weaning cornstarch resulted in episodes of hypoglycemia with blood glucoses range 50–60 mg/dl. Therefore, Diazoxide was started as the first line of therapy at age 4. Due to extensive hypertrichosis due to diazoxide she was switched to LAR octreotide three months later, initially at 10 mg monthly based on prior experience of her attending physician with other cases of hyperinsulinism of infancy. Dose raised to 10 mg every 21 days as she was having hypoglycemia with weaning feeds overnight on the monthly dose. Patient was then weaned off overnight feeds, was able to maintain blood glucose values > 70 mg/dl in the range of 80–90 mg/dl and an 8 hour or longer overnight fast. No tumors to date. At age 6 years and 7 months old she was weaned off the Octreotide and she remains euglycemic with blood glucose values > 70 mg/dl, up to 6 months later.

Patient didn’t develop any side effects while receiving the LAR; no gallstones or sludge and she maintained normal linear growth and weight. IGF-1 level was not measured since her linear growth remained appropriate with height range 50-60th percentile.

## Discussion

The incidence of hypoglycemia in BWS is about 50% with 20% of cases reported to be prolonged and difficult to control [4]. In about 4% of BWS patients have hypoglycemia that extends beyond one month of age and requires intensive medical management and even surgery [11]. It is important to treat the hyperinsulinemic hypoglycemia early and effectively, as it may pose a significant impact on neurocognitive development [4]. The two cases we describe present severe cases with hypoglycemia that persists to a later age than most BWS cases with hypoglycemia [3]. Both had hypoketotic hypoglycemia with relative hyperinsulinemia, with unsuppressed insulin levels while severely hypoglycemic, as was described previously [12].

The exact cause of hyperinsulinism in BWS is unclear. The molecular etiology of BWS itself is complex, involving alterations in two imprinting centers on chromosome 11p15.5 that affect the expression of IGF2 and/or tumor suppression genes. The ABCC8 and KCNJ11 genes, which encode the two components of the pancreatic β-cell ATP-sensitive potassium channel, are present on chromosome 11p15.1, in close proximity to the BWS locus, and are the most common cause for hyperinsulinemic hypoglycemia of infancy [13]. The K-ATP channel is a hetero-octameric complex composed of four Kir6.2 subunits and four high-affinity SUR1 subunits. The most common causes of diffuse congenital hyperinsulinism are the autosomal recessive and dominant mutations in ABCC8 and KCNJ11 genes, and when the mutation fully abolishes the function of the K-ATP channel the patients do not respond to diazoxide [5,13]. Milder autosomal dominant mutations may respond to diazoxide. Focal disease is always sporadic and has a distinctive genetic etiology involving two independent events – inheritance of a paternal mutation in ABCC8 or KCNJ11 and somatic loss of the maternal 11p allele (11p15.1 to 11p15.5) involving the ABCC8 and KCNJ11 region within the focal lesion. The loss of the maternal allele unmasks the paternally inherited K-ATP channel mutation in addition to an imbalance in the imprinted genes in this region (maternally expressed tumor suppressor genes H19 and CDKN1C, and the paternally expressed growth factor IGF2). An imbalance of the 11p15 region, which is close to the BWS locus, promotes proliferation of β-cells that evolve into a focal adenomatous hyperplasia [5,13].

In a thorough molecular analysis of 200 cases of BWS, Cooper et al. [3] found no significant differences between BWS molecular subgroups and the frequencies of neonatal hypoglycemia (overall frequency = 54%). Though mutations in *ABCC8 and KCNJ11* have not been implicated directly as the cause of hyperinsulinism in BWS, including a thoroughly investigated case by Hussain et al. [5], it is reasonable to speculate that their proximity to the imprinting centers associated with BWS, may affect their expression. Similarly, the insulin gene itself (INS) is located at 11p15.5, close to the imprinting centers implicated in BWS. It is interesting that both focal and diffuse forms of hyperinsulinism have been noted in BWS as confirmed by PET scans ahead of surgery [14] similar to the experience in *ABCC8* and *KCNJ11*.

Aynsley-Green et al. outlined the management of hypoglycemia due to hyperinsulinism of infancy in general, and recommended the use of parenteral agents if oral treatment with diazoxide fails [15]. Diazoxide is a K-ATP channel agonist. It requires a functional channel to be effective. It is considered the first line of pharmacological therapy. However, diazoxide has undesirable side effects, especially hypertrichosis and at higher doses, salt and water retention leading to cardiopulmonary congestion. Similarly, the approaches to treatment of hypoglycemia in Children with BWS include the use of diazoxide, octreotide and surgery in severe refractory cases. High glucose infusion rates may be needed to maintain euglycemia initially.

Octreotide is considered the second line of treatment. It inhibits insulin secretion distal to the K-ATP channel by inducing hyperpolarization of β-cells and direct inhibition of voltage dependent calcium channel. Gerver described the successful use of the short acting somatostatin analogue on the metabolism of an infant with BWS with hyperinsulinemic hypoglycemia, where the development of water retention secondary to Diazoxide necessitated switching to Octreotide [10]. To our knowledge no cases were published reporting the use of the depot form in BWS. Both of the cases we report here were tried on diazoxide and were switched to multiple daily injection of octreotide due to suboptimal control and/or adverse effects. After response to the short acting formulation of octreotide was demonstrated the two cases were successfully converted to once monthly injection with LAR. In the second case, she was taken off 4 times daily octreotide injections and managed with very frequent feeding resulting on excessive weight gain. The latter outcome may have been prevented had she been tried on the depot formulation earlier. The LAR form is typically given as a monthly injection. However, in our second case the patient required continuous feeds overnight to maintain euglycemia, and therefore the LAR frequency (and effective dose) was increased to every 21 days without any complications*.*

In some cases with BWS unresponsive to medical therapy, surgical intervention with partial or total pancreatectomy must be considered. Very few such cases have been described in the literature. Of note, both diffuse and focal forms were recently reported in BWS infants, when preoperative PET scan was utilized to help guide the surgery [11]. It is difficult to differentiate focal vs. diffuse lesions based on clinical grounds and diagnosis requires the use of Molecular imaging with [18 F]- L- 3,4-dihydroxyphenylalanine ([18 F]- DOPA) PET [13,14]. Unfortunately, this modality is only available in a limited number of institutions around the world and in the United States it is presently only available at Children’s Hospital of Philadelphia and Cook Children’s Hospital in Texas. It is particularly beneficial to identify focal lesions, as partial pancreatectomy is curative [13,14] and avoids the frequent development of diabetes mellitus in cases undergoing near-total pancreatectomy.

## Conclusion

There is limited data on the use of parenteral octreotide in BWS. The two genetically confirmed cases of BMS hereby reported have each been successfully managed on LAR for over two years and the treatment has been well tolerated. Both demonstrated an excellent response to the LAR, achieving euglycemia, making it a useful option in BWS patients with severe hypoglycemia due to hyperinsulinism, that cannot tolerate or do not respond to diazoxide.

## Consent

Written informed consent was obtained from the patients for publication of these case reports. A copy of the written consent is available for review by the Editor-in-Chief of this journal.

## Competing interests

No competing or conflicts of interests.

## Authors’ contributions

All authors took part in the clinical care of the subjects, and in the writing of the manuscript. All authors read and approved the final manuscript.
